# Numerical Simulation of Stresses in Functionally Graded HCS-MgO Cylinder Using Iterative Technique and Finite Element Method

**DOI:** 10.3390/ma15134537

**Published:** 2022-06-28

**Authors:** Sandeep Kumar Paul, Parth Dinesh Mehta, Manoj Sahni, Ernesto León-Castro

**Affiliations:** 1 Department of Mathematics, School of Technology, Pandit Deendayal Energy University, Gandhinagar 382426, Gujarat, India; sandeeppaulkumar@gmail.com (S.K.P.); mehtaparths@gmail.com (P.D.M.); 2 Department of Management, Faculty of Economics and Administrative Sciences, Universidad Católica de la Santísima Concepción, Concepción 4030000, Chile; eleon@ucsc.cl

**Keywords:** functionally graded material (FGM), pressure vessel, Young’s modulus, finite element method (FEM), stress–strain

## Abstract

In this study, a thick hollow axisymmetric functionally graded (FG) cylinder is investigated for steady-state elastic stresses using an iteration technique and the finite element method. Here, we have considered a functionally graded cylinder tailored with the material property, namely, Young’s modulus, varying in an exponential form from the inner to outer radius of the cylinder. A mathematical formulation for stress analysis of functionally graded cylinder under internal and external pressure conditions is developed using constitutive relations for stress–strain, strain–displacement relations and the equation of equilibrium. The effect of the in-homogeneity parameter on radial displacement, radial and tangential stresses in a functionally graded cylinder made up of a High Carbon Steel (HCS) metal matrix, reinforced with Magnesium Oxide (MgO) ceramic is analyzed. The iterative method implemented is fast and converges to the solution which can be further improved by considering a higher number of iterations. This is depicted graphically by using radial displacement and stresses in a pressurized functionally graded cylinder obtained for the first two iterations. An iterative solution for non-FGM (or homogeneous material) is validated using the finite element method. The mechanical responses of the functionally graded cylinder obtained from the iterative method and the finite element method are then compared and found to be in good agreement. Results are presented in graphical and tabular form along with their interpretations.

## 1. Introduction

Functionally graded materials (FGMs) are a new class of advanced and innovative materials and are usually made up of metals and ceramics. There are several structural components with a cylindrical shape, which are commonly used in many engineering applications such as pressure vessels, submarine, boiler, gun pipe, drive shaft, CNG storage cylinder etc. Due to the sharp interface in the traditional composite material a serious drawback of delamination was observed by Japanese material scientists during the aerospace project in 1984 [1]. This material failure was one of the major causes that led to the development of functionally graded materials (FGMs). Basically, functionally graded materials are non-homogeneous engineering materials in which a sharp interface can be changed with a smooth transitioning interface that reduces stress concentration at the interface and avoid the occurrence of material failure. The study on the dynamic behavior of a viscoelastic hollow FGM cylinder under thermomechanical loads is done using the local Petrov–Galerkin method by Akbari et al. [2]. Azad et al. [3] investigated non-linear higher order differential equation using a new technique combining the perturbation and harmonic balance methods, and obtained good results even in strong non-linearity. Two rotating FG axisymmetric disks are discussed; one with uniform thickness and the other following a hyperbolic or parabolic convergent profile for thickness. The mechanical responses for both disks are compared by Bayat et al. [4]. The authors indicate that the thickness profile along parabolic or hyperbolic convergent modes is more efficient than uniform thickness in the designing application of rotary disks. The numerical solution for a disk made of metal matrix composite such as Al-SiC is carried out using ANSYS^®^ and the results are compared with an analytical solution by Bektaş and Akça [5]. Two-dimensional finite element and generalized differential quadrature methods are used to study the response of free vibration in FGM plates and cylinders and the result is compared with three dimensional analytical solutions by Brischetto et al. [6]. Chauhan and Srivastava [7] have discussed iterative schemes for several types of Runge–Kutta methods to find the numerical solution of ordinary differential equations. Wave propagation for a functionally graded (FG) circular cylinder made of Ni and $Al_{2}O_{3}$ is discretized using the finite difference method (FDM) under dynamic loads and the power law material variation in a radial direction [8]. Ebrahimi and Najafizadeh [9] have worked on the free vibration analysis of a 2D FG cylindrical shell where boundary conditions and spatial derivatives are discretized using generalized integral quadrature and generalized differential quadrature methods. An exponentially graded rotating thick cylinder is investigated for elastic analysis using the Frobenius method by Gharibi et al. [10]. Using the homotopy perturbation method, an FGM annular rotating disk is investigated for elasticity and plasticity analysis under plane stress conditions where the thickness of the disk and densities are varying in the radial direction [11]. Applying 3D finite element analysis by using commercial software ABAQUS, the influence of material parameters on the response of composite steel-concrete beam is studied by Jaafer and Kareem [12]. An axisymmetric elastic stress analysis is performed for a functionally graded spinning disk where thickness and material properties of the rotary disk vary along the power law function, and the numerical solution is obtained by applying the finite difference method under clamped-clamped, clamped-free, and free-free boundary conditions [13]. Kacar [14] has derived an elastic analytical solution for an FGM disk, cylinder and sphere, where material changes by power law in the radial direction. Using the energy principle and Lagrange equations, a dynamic model of structure is investigated assuming combined dynamic properties to obtain an optimum design [15]. Researchers [16,17] have analysed thermo-mechanical stresses in a functionally graded rotating disk and it is observed that a disk made up of functionally graded material is a better option than composite materials. An FGM hollow cylinder formed of alumina (ceramic) and nickel (metal) is studied as an unsteady state thermo-mechanical problem due to the change in point heat source subjected to convective heat transfer [18]. The optimization of several functionally graded structures, such as rotating disk, tubes, plates, spheres and cylinders, are discussed by Nikbakht et al. [19]. The authors of [20,21,22] have studied 2D mechanical problems for functionally graded materials with cylindrical and spherical geometry. The outcome of these investigations helps in understanding and tailoring the design parameters of functionally graded materials. Singh and Sahni [23] have carried out an analytical solution for the displacement and stresses of innovative composite material made of FGM where material gradation and thickness change along the radial dimension. Zafarmand and Hassani [24] have used the graded finite element method to solve displacement and stress equations in radial and axial directions for solid and annular disks whereas Emilio Martínez-Pañeda [25] studied the performance of functionally graded properties using FEM. The elastic properties at macroscopic variation, inherent to functionally graded materials (FGMs), were introduced at the element level by means of nodal based gradation, often via an auxiliary (non-physical) temperature-dependence and a Gauss integration point based gradation. Sahni et al. [1] presented a secondary creep stress–strain analysis of a pressurized cylinder made up of FGM and demonstrated the effect of varying the volume reinforcement of ceramic in a metal matrix on creep stresses and strains in a functionally graded rotating cylinder. In this study, an axisymmetric thick hollow FGM cylinder is considered, which is made up of High Carbon Steel (HCS) as the inner, and magnesium oxide (MgO) as the outer, material. The Poisson’s ratio is invariant and Young’s modulus varies with the exponential law along the radial direction. This work is based on steady state and plain strain conditions under uniform internal and external pressure conditions. The results for displacement and stresses are carried out by a simple iterative technique and are then compared with the results obtained by the finite element method. A good agreement is found between these two methods with a small acceptable error. A solution for stresses and displacement can be improved by considering a greater number of iterations.

## 2. Mathematical Formulation of Stress Analysis

**Figure 1:**
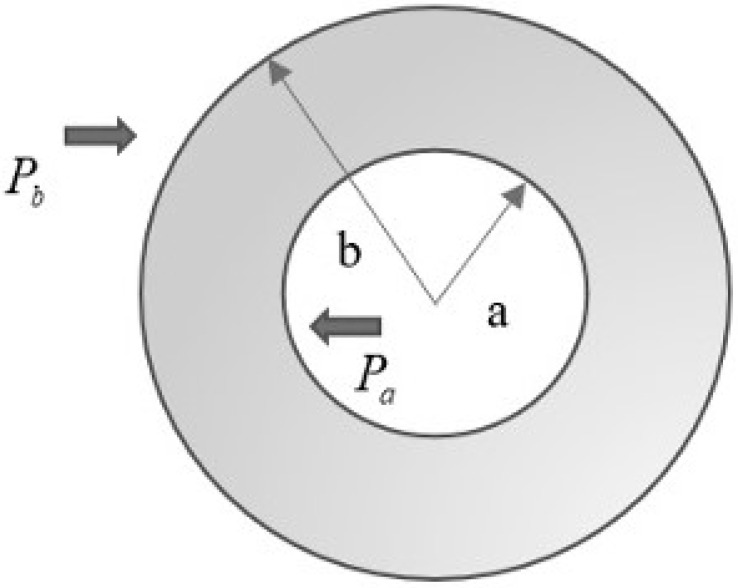
Axisymmetric functionally graded cylinder with inner radius *a*, outer radius *b* and under internal and external pressure, $P_{a}$ and $P_{b}$, respectively.

In this problem, an axisymmetric thick hollow cylinder, presented in Figure 1, is considered under a steady state plane strain condition with inner and outer radii as *a* and *b* respectively. Uniform internal and external pressures at the inner and outer surfaces of the cylinder are denoted by $P_{a}$ and $P_{b}$, respectively. Poisson’s ratio ν is kept constant and the modulus of elasticity E(r) varies along the radial direction that follows exponential law, given as [26]:

(1) $$E\left(r\right)=E_{a}e^{m\left(r-a\right)},$$

where $m=\frac{1}{b-a}\log\left(\frac{E\left(b\right)}{E\left(a\right)}\right)$. Here, $E\left(a\right)$ and $E\left(b\right)$ are constants of Young’s modulus at inner and outer radii, respectively, and *m* is the material gradation parameter.

**Figure 2:**
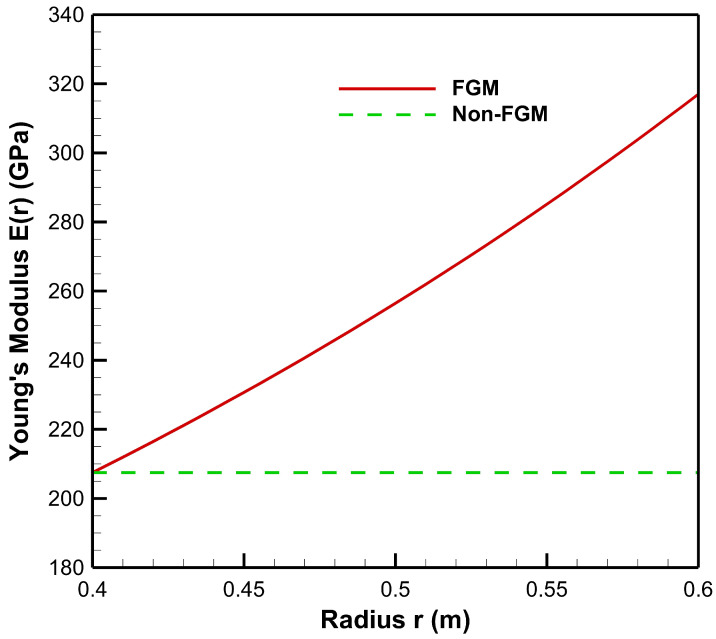
Variation of Young’s modulus along the radius of the cylinder.

Figure 2 presents the variation of Young’s modulus in FGM and non-FGM cylinders. The Young’s modulus for the FGM cylinder is continuously increasing under an exponential profile given by Equation (1).

Using Navier’s equation in the radial direction for an axisymmetric functionally graded cylinder can be given as [5]:

(2) $$\frac{d\sigma_{r}}{dr}+\frac{\sigma_{r}-\sigma_{\theta }}{r}=0,$$

where $\sigma_{r}$ and $\sigma_{\theta }$ are radial and tangential stresses, respectively. From Hooke’s law for isotropic material [10], we have:

(3) $$\sigma_{r}=E\left(r\right)\left(\lambda \frac{du}{dr}+\delta \frac{u}{r}\right)\;\mathrm{and}\;\sigma_{\theta }=E\left(r\right)\left(\delta \frac{du}{dr}+\lambda \frac{u}{r}\right),$$

where radial displacement *u* is the function of radius, *r*. Lame’s parameters, δ and λ, can be given [26] as:

(4) $$\lambda =\frac{\left(1-\nu \right)}{\left(1+\nu \right)\left(1-2\nu \right)}\;\mathrm{and}\;\delta =\frac{\nu }{\left(1+\nu \right)\left(1-2\nu \right)}.$$

Under the plane strain condition, strain–displacement relation [26] can be given as:

(5) $$\epsilon_{r}=\frac{du}{dr}\;\mathrm{and}\;\epsilon_{\theta }=\frac{u}{r}.$$

The pressure boundary conditions prescribed at inner and outer radii are defined as:

(6) $$\sigma_{r}\left(a\right)=-P_{a}\;\mathrm{and}\;\sigma_{r}\left(b\right)=-P_{b}.$$

Using Equations (1)–(5), Navier’s equation can be expressed in radial displacement as:

(7) $$r^{2}\frac{d^{2}u}{dr^{2}}+r\frac{du}{dr}-u=-mr^{2}\frac{du}{dr}-\frac{m\nu }{1-\nu }ru.\;$$

### Iterative Solution Technique

Applying a simple iterative method [26] on Equation (7), the governing differential equation can be written as:

(8) $$r^{2}\frac{d^{2}u_{n+1}}{dr^{2}}+r\frac{du_{n+1}}{dr}-u_{n+1}=-mr^{2}\frac{du_{n}}{dr}-\frac{m\nu }{1-\nu }ru_{n}\;,\;\mathrm{where}\;n\;=\;0,\;1,\;2,\;\ldots \;\ldots$$

The linear and non-linear parts from Equation (8) can be written as:

(9) $$L\left(u_{n}\left(r\right)\right)=r^{2}\frac{d^{2}u_{n}}{dr^{2}}+r\frac{du_{n}}{dr}-u_{n}\;\mathrm{and}\;N\left(u_{n}\left(r\right)\right)=-mr^{2}\frac{du_{n}}{dr}-\frac{m\nu }{1-\nu }ru_{n}.$$

The initial solution can be obtained by solving Equation (9) as:

(10) $$L\left(u_{o}\left(r\right)\right)=0\;\mathrm{with}\;B.C:{\left(\lambda \frac{du_{o}}{dr}+\delta \frac{u_{o}}{r}\right)}_{a}=-\frac{P_{a}}{E\left(a\right)}\;\mathrm{and}\;{\left(\lambda \frac{du_{o}}{dr}+\delta \frac{u_{o}}{r}\right)}_{b}=-\frac{P_{b}}{E\left(b\right)}.$$

The general solution of Equation (10) can be expressed as:

(11) $$u_{o}\left(r\right)=\frac{C_{1}}{r}+C_{2}r.$$

On applying boundary conditions using Equation (10) on Equation (11), we obtain constants of integration, namely $C_{1}$ and $C_{2}$ given as:

$C_{1}=\frac{\left(P_{b}e^{-m\left(b-a\right)}-P_{a}\right)a^{2}b^{2}}{E_{a}\left(\delta -\lambda \right)\left(b^{2}-a^{2}\right)}$ and $C_{2}=-\frac{1}{E_{a}\left(\lambda +\delta \right)}\left(P_{a}+\frac{\left(P_{b}e^{-m\left(b-a\right)}-P_{a}\right)b^{2}}{\left(b^{2}-a^{2}\right)}\right)$.

The first iteration can be obtained by solving Equation (8) for *n* = 0 as:

(12) $$r^{2}\frac{d^{2}u_{1}}{dr^{2}}+r\frac{du_{1}}{dr}-u_{1}=-mr^{2}\frac{du_{0}}{dr}-\frac{m\nu }{1-\nu }ru_{0},$$

with boundary conditions given as:

(13) $$E\left(a\right)\left(\lambda {\left(\frac{du_{1}}{dr}\right)}_{a}+\delta {\left(\frac{u_{1}}{r}\right)}_{a}\right)=-P_{a}\;\mathrm{and}\;E\left(b\right)\left(\lambda {\left(\frac{du_{1}}{dr}\right)}_{b}+\delta {\left(\frac{u_{1}}{r}\right)}_{b}\right)=-P_{b}.$$

Solving Equation (12), we get the solution for first iteration $u_{1}$ as:

(14) $$u_{1}=\frac{C_{3}}{r}+C_{4}r-\frac{m\left(1-2\nu \right)}{\left(1-\nu \right)}C_{1}-\frac{m}{3\left(1-\nu \right)}C_{2}r^{2}.$$

Applying boundary condition (13) on Equation (14), we get constants of integration $C_{3}$ and $C_{4}$ as:

(15) $$C_{3}=\frac{a^{2}b^{2}}{\left(\delta -\lambda \right)\left(a+b\right)}\left(\frac{-2\lambda mC_{2}}{3\left(1-\nu \right)}-\frac{m\delta C_{2}}{3\left(1-\nu \right)}+\frac{\delta mC_{1}\left(1-2\nu \right)}{ab\left(1-\nu \right)}+\frac{P_{b}e^{m\left(b-a\right)}-P_{a}}{E_{a}\left(b-a\right)}\right)$$

and

(16) $$\begin{array}{cc} C_{4}= & \frac{1}{\left(\lambda +\delta \right)}\left[\frac{2\lambda mbC_{2}}{3\left(1-\nu \right)}+\frac{mb\delta C_{2}}{3\left(1-\nu \right)}+\frac{m\left(1-2\nu \right)C_{1}}{b\left(1-\nu \right)}-\frac{P_{b}e^{-m\left(b-a\right)}}{E_{a}}\right.\\ - & \left.\frac{a^{2}}{a+b}\left(\frac{-2m\lambda C_{2}}{3\left(1-\nu \right)}-\frac{m\delta C_{2}}{3\left(1-\nu \right)}+\frac{m\delta \left(1-2\nu \right)C_{1}}{ab\left(1-\nu \right)}+\frac{P_{b}e^{-m\left(b-a\right)}-P_{a}}{E_{a}\left(b-a\right)}\right)\right]. \end{array}$$

On repeating the above process on Equation (8) for n=1, i.e.:

(17) $$r^{2}\frac{d^{2}u_{2}}{dr^{2}}+r\frac{du_{2}}{dr}-u_{2}=-mr^{2}\frac{du_{1}}{dr}-\frac{m\nu }{1-\nu }ru_{1}$$

and considering the boundary condition given as:

(18) $$E\left(a\right)\left(\lambda {\left(\frac{du_{2}}{dr}\right)}_{a}+\delta {\left(\frac{u_{2}}{r}\right)}_{a}\right)=-P_{a}\;\mathrm{and}\;E\left(b\right)\left(\lambda {\left(\frac{du_{2}}{dr}\right)}_{b}+\delta {\left(\frac{u_{2}}{r}\right)}_{b}\right)=-P_{b},$$

we get the second iteration $u_{2}$ as:

(19) $$u_{2}=C_{6}r+\frac{C_{5}}{r}-\frac{mC_{4}}{3\left(1-\nu \right)}r^{2}+\frac{m\left(2\nu -1\right)C_{3}}{\left(1-\nu \right)}-\frac{m^{2}\left(\nu -2\right)C_{2}}{24{\left(1-\nu \right)}^{2}}r^{3}+\frac{\nu m^{2}\left(1-2\nu \right)C_{1}}{2{\left(1-\nu \right)}^{2}}r\log\left(r\right),$$

where constants of integration $C_{5}$ and $C_{6}$ are obtained by using Equation (18) for the boundary condition. Thus, $C_{5}$ and $C_{6}$ are evaluated as:

(20) $$\begin{array}{cc} C_{5} & =\frac{a^{2}b^{2}}{\left(a+b\right)\left(\delta -\lambda \right)}\left[\frac{-m\left(2\lambda +\delta \right)C_{4}}{3\left(1-\nu \right)}-\frac{m^{2}\left(3\lambda +\delta \right)\left(a+b\right)\left(\nu -2\right)C_{2}}{24{\left(1-\nu \right)}^{2}}-\frac{m\delta \left(2\nu -1\right)C_{3}}{ab\left(1-\nu \right)}\right.\\ & +\left.\frac{m^{2}\nu \left(\lambda +\delta \right)\left(1-2\nu \right)C_{1}}{2\left(b-a\right){\left(1-\nu \right)}^{2}}\log\left(\frac{b}{a}\right)+\left.\frac{P_{b}e^{-m\left(b-a\right)}-P_{a}}{E_{a}\left(b-a\right)}\right]\right. \end{array}$$

and

(21) $$\begin{array}{cc} C_{6} & =\frac{1}{\left(\delta +\lambda \right)}\left[\frac{2\lambda mbC_{4}}{3\left(1-\nu \right)}+\frac{\lambda b^{2}m^{2}\left(\nu -2\right)C_{2}}{8{\left(1-\nu \right)}^{2}}-\frac{m^{2}\lambda \nu \left(1-2\nu \right)C_{1}}{2{\left(1-\nu \right)}^{2}}\left(1+\log b\right)\right.\\ & -\left.\frac{\delta m\left(2\nu -1\right)C_{3}}{b\left(1-\nu \right)}+\frac{\delta b^{2}m^{2}\left(\nu -2\right)C_{2}}{24{\left(1-\nu \right)}^{2}}+\frac{\delta mbC_{4}}{3\left(1-\nu \right)}-\frac{\delta \nu m^{2}\left(1-2\nu \right)C_{1}}{2{\left(1-\nu \right)}^{2}}\log b-\frac{P_{b}e^{-m\left(b-a\right)}}{E_{a}}\right.\\ & +\left.\frac{a^{2}}{a+b}\left(\frac{m\left(2\lambda +\delta \right)C_{4}}{3\left(1+\nu \right)}+\frac{m^{2}\left(3\lambda +\delta \right)\left(a+b\right)\left(\nu -2\right)C_{2}}{24{\left(1-\nu \right)}^{2}}+\frac{m\delta \left(2\nu -1\right)C_{3}}{ab\left(1-\nu \right)}\right.\right.\\ & -\left.\left.\frac{\left(\lambda +\delta \right)m^{2}\nu \left(1-2\nu \right)C_{1}}{2\left(b-a\right){\left(1-\nu \right)}^{2}}\log\left(\frac{b}{a}\right)-\frac{P_{b}e^{-m\left(b-a\right)}-P_{a}}{E_{a}\left(b-a\right)}\right)\right] \end{array}$$

Since the remaining terms are too long to present here, we stop at this point. However, by considering a greater number of iterations, the accuracy of the solution can be further improved. Considering $u_{2}\left(r\right)$ as a second iteration in the displacement and substituting $u\left(r\right)=u_{2}\left(r\right)$ in Equations (3) and (5), we obtain stresses along radial direction as:

(22) $$\begin{array}{cc} \sigma_{r}\left(r\right)= & \frac{E_{a}e^{m\left(r-a\right)}}{\left(1+\nu \right)\left(1-2\nu \right)}\left[\left(1-\nu \right)\left(-\frac{C_{5}}{r^{2}}+C_{6}-\frac{2mC_{4}}{3\left(1-\nu \right)}r-\frac{m^{2}\left(\nu -2\right)C_{2}}{8{\left(1-\nu \right)}^{2}}r^{2}\right.\right.\\ + & \left.\frac{\nu m^{2}\left(1-2\nu \right)C_{1}}{2{\left(1-\nu \right)}^{2}}\left(1+\log\left(r\right)\right)\right)+\nu \frac{C_{5}}{r^{2}}+\nu C_{6}+\frac{\nu m\left(2\nu -1\right)C_{3}}{\left(1-\nu \right)r}-\frac{\nu mC_{4}}{3\left(1-\nu \right)}r\\ + & \left.\frac{\nu m^{2}\left(\nu -2\right)C_{2}}{24{\left(1-\nu \right)}^{2}}r^{2}+\frac{\nu^{2}m^{2}\left(1-2\nu \right)C_{1}}{2{\left(1-\nu \right)}^{2}}\log\left(r\right)\right] \end{array}$$

and

(23) σθr=Eaemr−a1+ν1−2νν−C5r2+C6−2mC431−νr−m2ν−2C281−ν2r2+νm21−2νC121−ν21+logr+1−νC5r2+C6+1−ν m2ν−1C31−νr−mC431−νr−m2ν−2C2241−ν2r2+νm21−2νC121−ν2logr.

Using Equations (5) and (19), radial and tangential strains can also be obtained.

## 3. Results and Discussion

We consider an axisymmetric functionally graded thick hollow cylindrical pressure vessel with inner radius a = 0.4 m and outer radius b = 0.6 m under pressure conditions of high internal–low external pressure (20–50 MPa) and low internal–high external (20–50 MPa) pressure. Here, Poisson’s ratios $\nu_{M}=0.295$ and $\nu_{C}=0.17$ are considered for inner (metal) and outer (ceramic) materials respectively. Poisson’s ratio ν for the FGM cylinder is kept constant and is considered an average of $\nu_{M}$ and $\nu_{C}$. The pressure vessel is made up of High Carbon Steel (HCS) as the inner material and magnesium oxide (MgO) as the outer material [27]. Young’s modulus at the inner radius is E(a)=207.5 GPa and at the outer radius is E(b)=317 GPa.

### Numerical Computation

**Figure 3:**
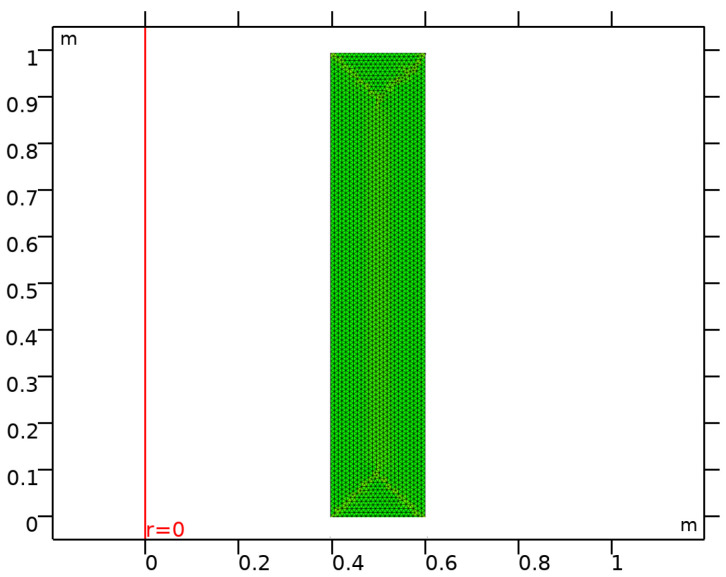
Finite element meshing of cross-section for an axisymmetric functionally graded cylinder.

In this study, results for radial stress, tangential stress and displacement are obtained numerically by using the FEM based solver COMSOL Multiphysics (5.4). The governing differential equation as given by Equation (3), under the prescribed boundary conditions given by Equation (8), is solved by using a solid mechanics module and axisymmetric geometric conditions. Using the global analytical function, Young’s modulus is defined over the domain of an axisymmetric functionally graded cylinder. As depicted in Figure 3, the domain of the cylinder is discretized with triangular elements into an extremely fine mesh by considering 5034 domain elements and 240 boundary elements. The model is then solved using a linear direct PARDISO solver and results for radial stress, tangential stress and displacement are obtained for a functionally graded cylinder with a relative tolerance of $10^{-5}$. Computation of stresses and displacement is also carried out for non-FGM material using a parametric sweep. The numerical solution obtained is then compared with an iterative solution for both FGM and non-FGM materials. Radial displacement and stresses have been solved up to two iterations using the iterative technique. After two iterations, a good agreement can be observed between the results obtained from iterative technique and FEM, which can further be improved by considering a greater number of iterations.

In Table 1, a comparison of the radial displacement in the FGM cylinder by FEM and iterative methods is shown, where the values in both methods are decreasing from the inner to the outer radius. Minimum and maximum absolute percentage errors of 2.82% and 4.03% occur at internal and external radii respectively. In Table 2, a comparison of radial stress in the FGM cylinder obtained by FEM and iterative methods is shown for different radial points. This table shows that radial stress decreases along the radius of the cylinder. At the internal radius, the absolute error is 0.04% and at the outer radius, an absolute error of 0.03 is observed; overall, % error along the radius of the cylinder is less than 1. Maximum error can be seen at radius r=0.525 which is 0.84%. Derived values of tangential stress for the FGM cylinder by FEM and iterative methods are shown for different radial points in Table 3. Tangential stress is decreasing from the inner to the outer radius of the FGM cylinder. From Table 3, we can observe that the propagation of error has a decreasing trend from inner to outer radial points. Maximum absolute error is obtained at the internal radius as 3.98% and the minimum error at the external radius is 1.93%.

**Figure 4:**
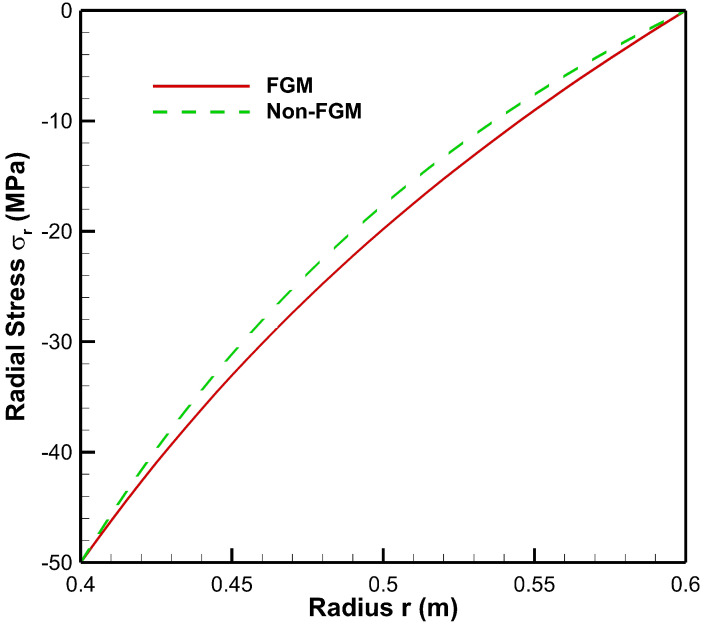
Radial Stress along the radius of the cylinder under internal pressure, $P_{a}=50$ MPa.

**Figure 5:**
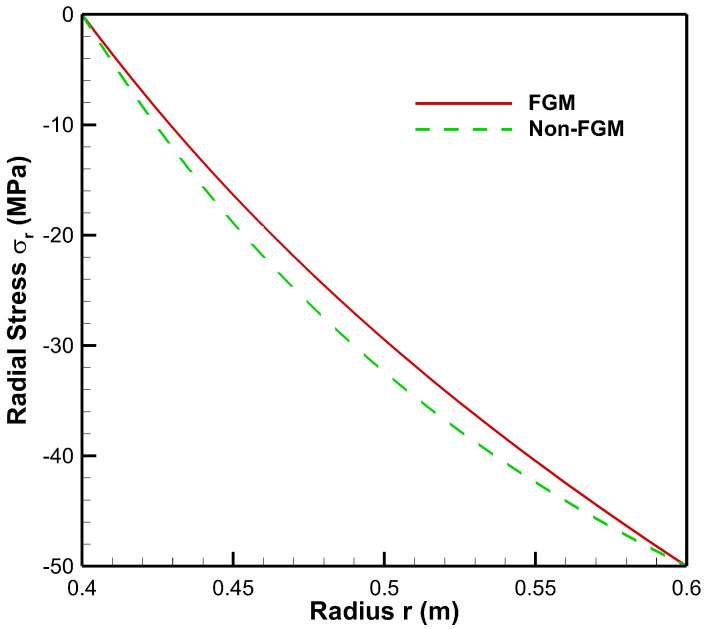
Radial Stress along the radius of the cylinder under external pressure, $P_{b}=50$ MPa.

**Figure 6:**
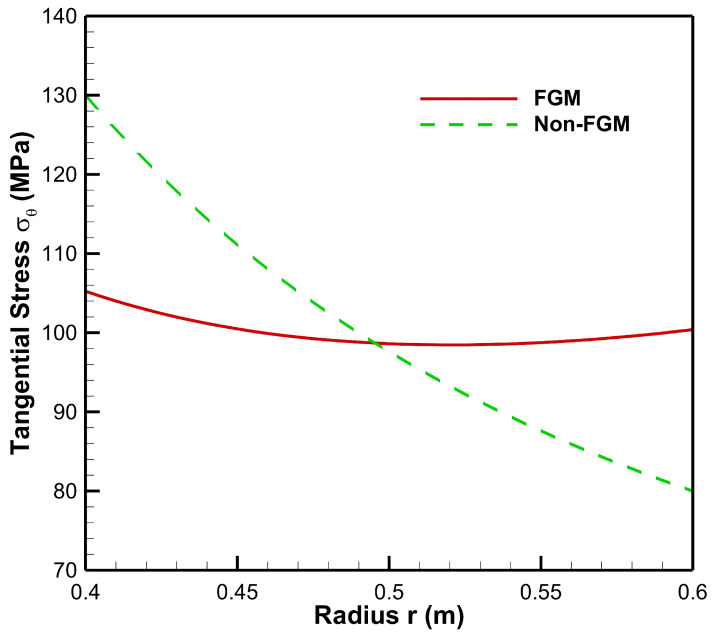
Tangential stress along the radius of the cylinder under internal pressure, $P_{a}=50$ MPa.

**Figure 7:**
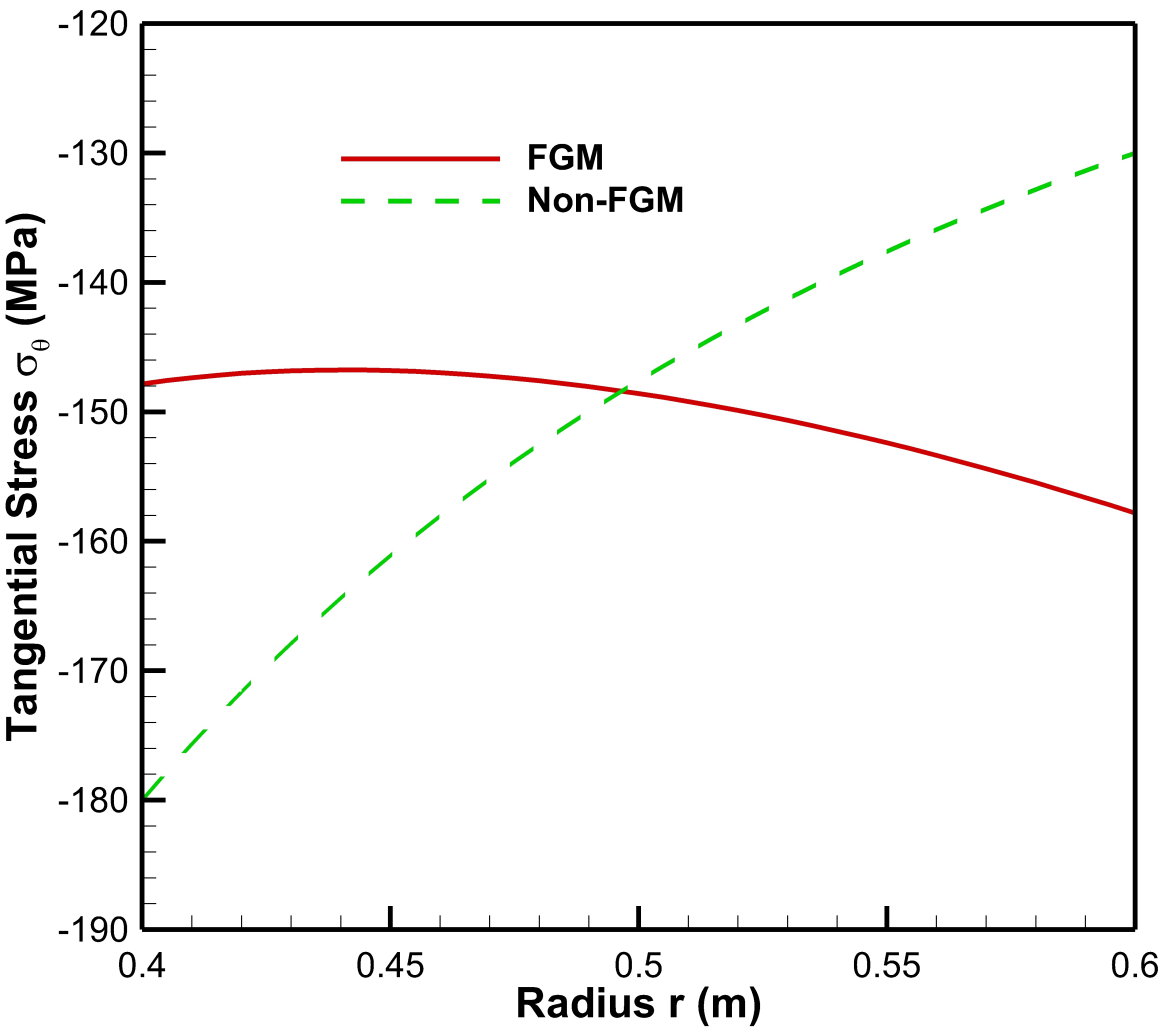
Tangential stress along the radius of the cylinder under external pressure, $P_{b}=50$ MPa.

**Figure 8:**
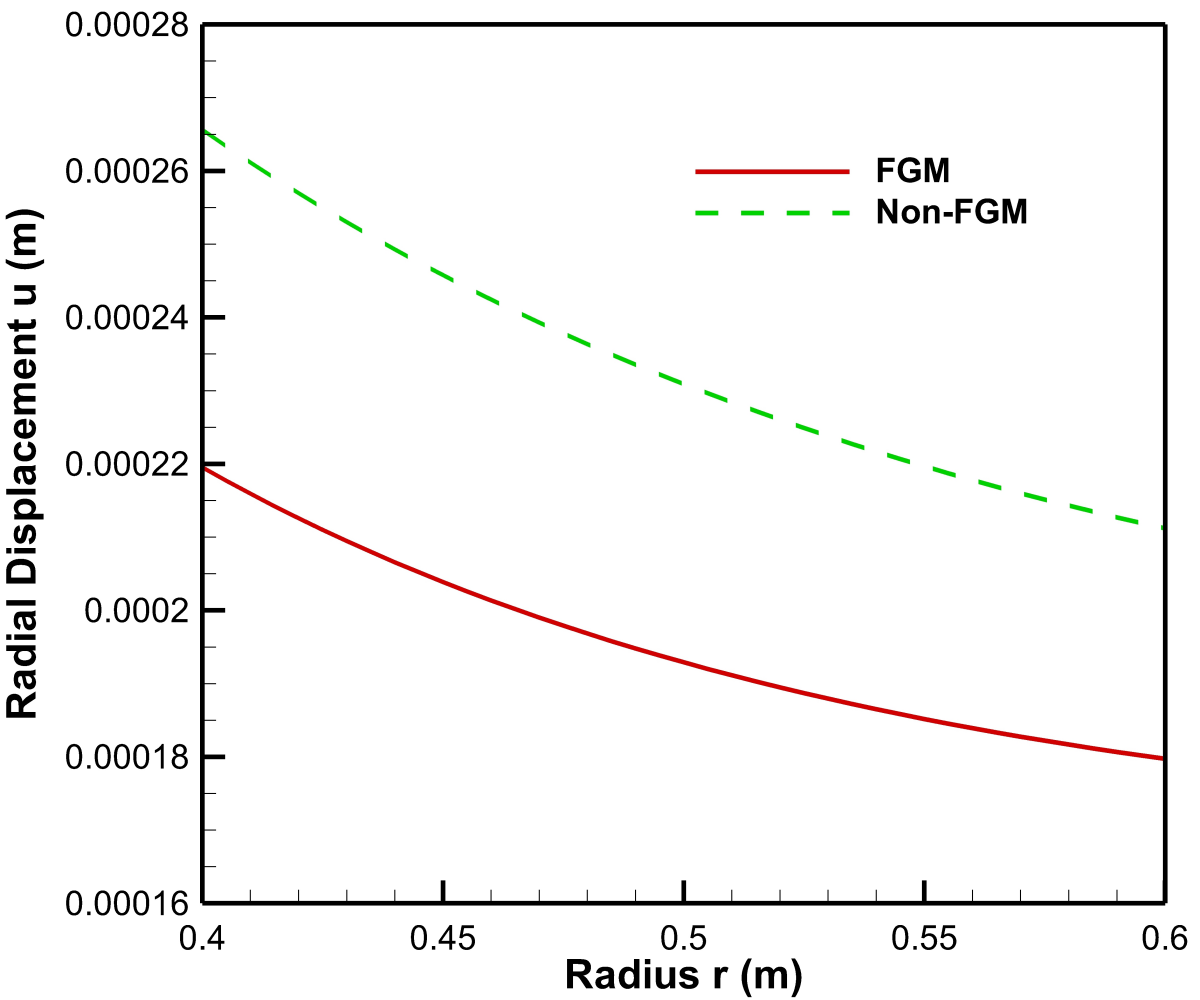
Radial displacement along the radius of the cylinder under internal pressure, $P_{a}=50$ MPa.

**Figure 9:**
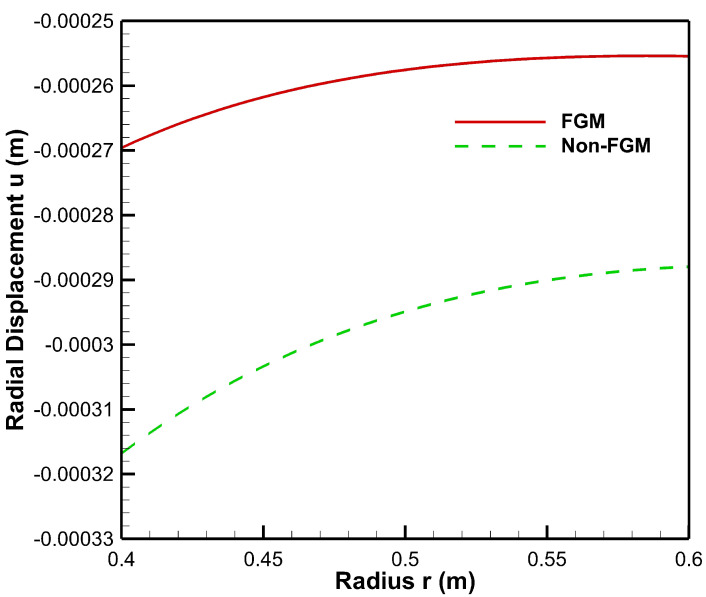
Radial displacement along the radius of the cylinder under external pressure, $P_{b}=50$ MPa.

Figure 4, Figure 5, Figure 6, Figure 7, Figure 8 and Figure 9 present stresses and displacement in FGM and non-FGM cylinders under internal/external pressure conditions obtained using the iterative technique. Figure 4 shows radial stress under internal pressure, $P_{a}=50$ MPa. Under the effect of internal pressure, radial stress in the FGM cylinder is higher in magnitude as compared to non-FGM. It can be observed due to higher internal pressure and increasing elasticity modulus from inner to outer radii of the FGM cylinder. It can be noted from Figure 4 that radial stress is on the higher side at the internal radial points of the cylinder due to low elasticity modulus at the inner radial points as compared to the outer radial points. Iterative radial stress in the cylinder under external pressure $P_{b}=50$ MPa is depicted in Figure 5. It can be observed that the magnitude of radial stress is lower in the FGM cylinder as compared to the non-FGM cylinder.

Figure 6 presents tangential stress in the cylinder under the influence of internal pressure $P_{a}=50$ MPa. As seen from Figure 6, tangential stress under internal pressure is tensile throughout the radius of the cylinder whereas from Figure 7, tangential stress under the effect of external pressure is found to be compressive throughout the radius of the cylinder. Moreover, under the internal pressure, the magnitude of tangential stress is found to decrease from inner to outer radii of the cylinder.

Tangential stress under internal pressure is on the higher side in the non-FGM cylinder but at outer radial points it is found to be higher for the FGM cylinder. Under the effect of external pressure, tangential stress in the FGM cylinder increases in magnitude towards the outer radius of the cylinder whereas in the non-FGM cylinder, tangential stress becomes less compressive as it moves along the outer radius of the cylinder.

As seen from Figure 8, radial displacement under the influence of internal pressure has tensile values for both FGM and non-FGM cylinders whereas radial displacement under the influence of external pressure exhibits the compressive behaviour, as shown in and Figure 9. Additionally, the magnitude of radial displacement in the case of the FGM cylinder is lower than that of the non-FGM cylinder under both internal and external pressure conditions as shown in Figure 8 and Figure 9. This behaviour can be attributed to the higher values of Young’s modulus for the FGM cylinder and the lower values for the non-FGM cylinder.

**Figure 10:**
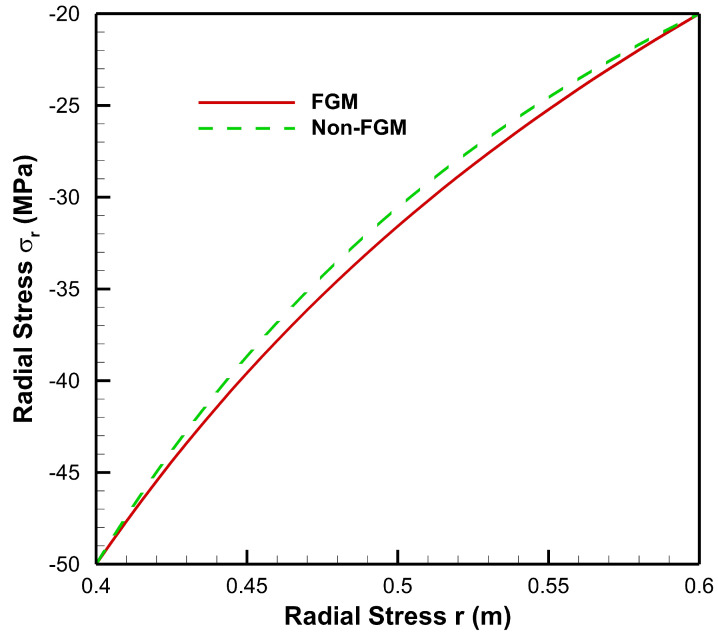
Radial Stress along the radius of the cylinder under internal pressure, $P_{a}=50$ MPa and external pressure, $P_{b}=20$ MPa.

In Figure 10, it can be observed that under the effect of high internal pressure and low external pressure, the magnitude of radial stress decreases from inner to outer radial points, for both FGM and non-FGM cylinders. However, for cylinders made up of High Carbon Steel–Magnesium Oxide functionally graded material, the magnitude of radial stress is found to be on the higher side as compared to the non-FGM (High Carbon Steel) cylinder. This mechanical response of the cylinder under high internal pressure and low external pressure is due to its material property, namely, Young’s modulus, which increases from inner to outer radii, causing a high resisting force due to which the radial stress generated is of a higher magnitude in comparison to the non-FGM cylinder.

**Figure 11:**
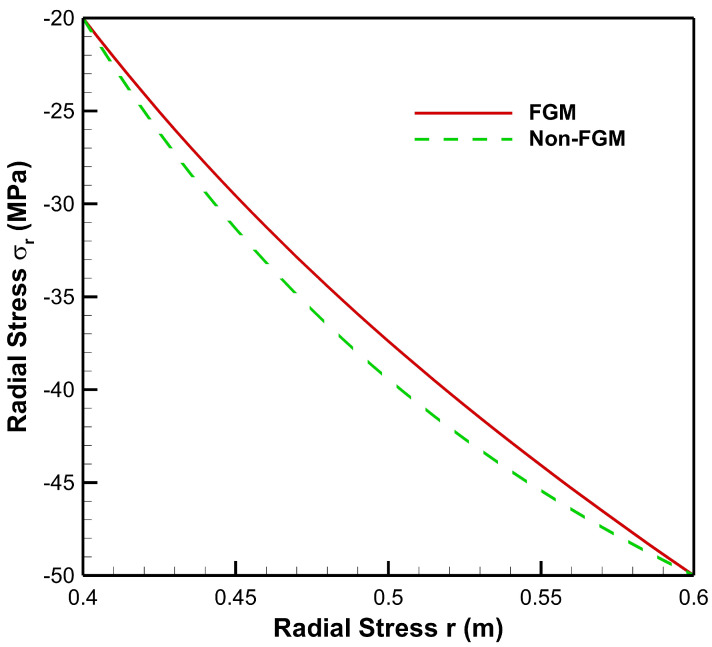
Radial Stress along the radius of the cylinder under internal pressure, $P_{a}=20$ MPa and external pressure, $P_{b}=50$ MPa.

As seen from Figure 11, under high external pressure and low internal pressure, radial stress for the FG cylinder is less as compared to the non-FGM cylinder, from internal to external radii of the cylinder. Hence, the amount of pressure required to move points from its original location is less for the FG cylinder. As the slope of radial stress decreases from inner to outer radii, this leads to producing a lower resistive force towards the outer radius of the functionally graded cylinder. This happens as Young’s modulus increases towards the outer radius; then the cylinder requires a lower resistive force to counter the external pressure. Hence, radial stress in the FG cylinder is lower in magnitude as compared to the non-FGM cylinder due to homogeneous Young’s modulus along the radial points.

**Figure 12:**
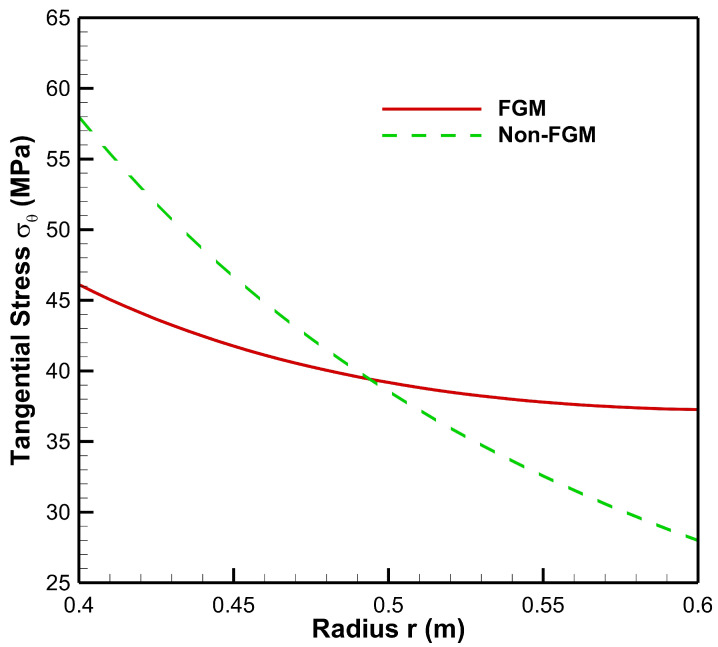
Tangential Stress along the radius of the cylinder under internal pressure, $P_{a}=50$ MPa and external pressure, $P_{b}=20$ MPa.

As seen from Figure 12, tangential stress from internal to external radii of the cylinder is found to be decreasing under high internal and low external pressure, but for the FGM cylinder, the magnitude of tangential stress is on the higher side at outer radial points as compared to the non-FGM cylinder. Tangential stresses under this case are found to be elastic throughout the radius of FGM and non-FGM cylinders.

**Figure 13:**
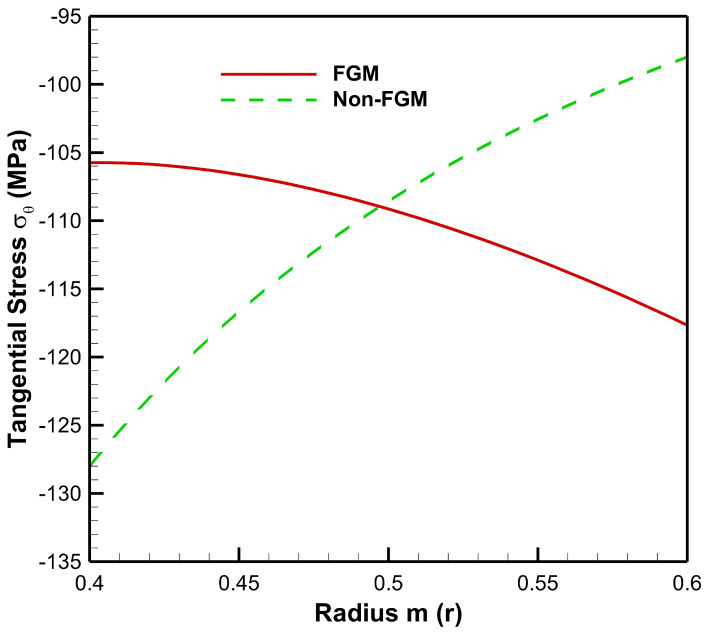
Tangential Stress along the radius of the cylinder under internal pressure, $P_{a}=20$ MPa and external pressure, $P_{b}=50$ MPa.

From Figure 13, under low internal and high external pressures, the magnitude of tangential stress for the FGM cylinder increases from inner to outer radii whereas in the non-FGM cylinder tangential stress is higher at inner radial points and decreases towards the outer radius of the cylinder.

It can be observed that the resistance force required by material points to move out from its external surface is less in the FGM cylinder as compared to the non-FGM cylinder and, hence, it saves a lot of stress.

**Figure 14:**
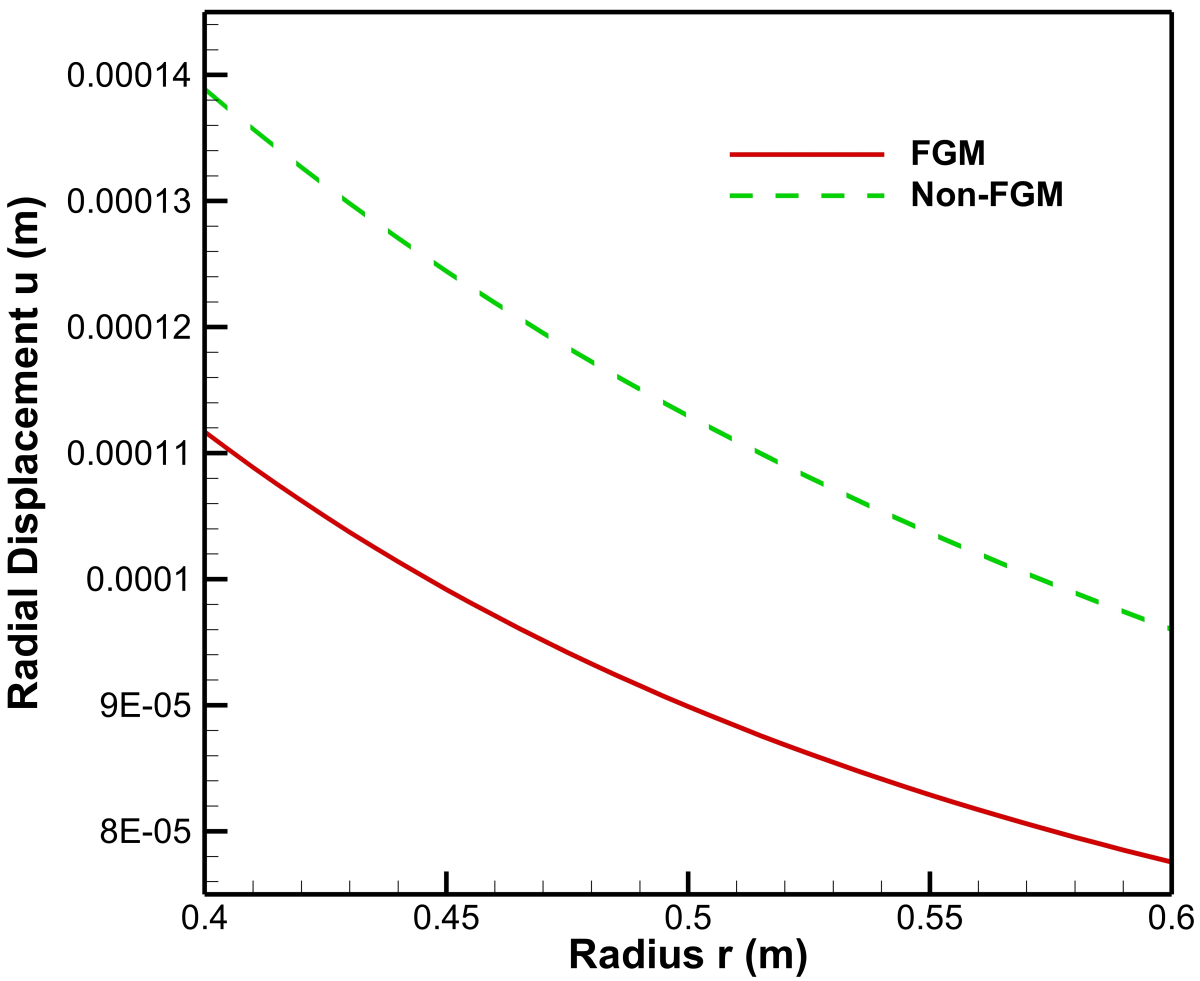
Displacement along the radius of the cylinder under internal pressure, $P_{a}=50$ MPa and external pressure, $P_{b}=20$ MPa.

From Figure 14, it can be observed that under high internal and low external pressure conditions, displacement decreases throughout the cylinder and is found to be lowest for the FGM cylinder. The high internal pressure causes compressive strains with displacement values decreasing along the radius of the cylinder. Due to graded elasticity in the FGM cylinder, it has a lower magnitude of stress as compared to the non-FGM cylinder. Moreover, displacement in both FGM and non-FGM cylinders is found to be compressive towards the outer radial points.

**Figure 15:**
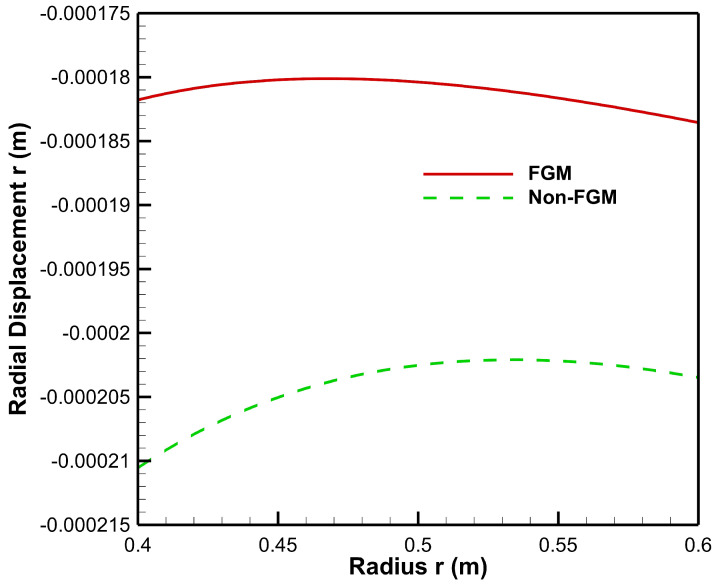
Displacement along the radius of the cylinder under internal pressure, $P_{a}=20$ MPa and external pressure, $P_{b}=50$ MPa.

Under low internal and high external pressure, as shown in Figure 15, displacement in the non-FGM cylinder has a higher magnitude than in the FGM cylinder and is also found to be increasing along the radius of the cylinder whereas it decreases in the case of the FGM cylinder.

**Figure 16:**
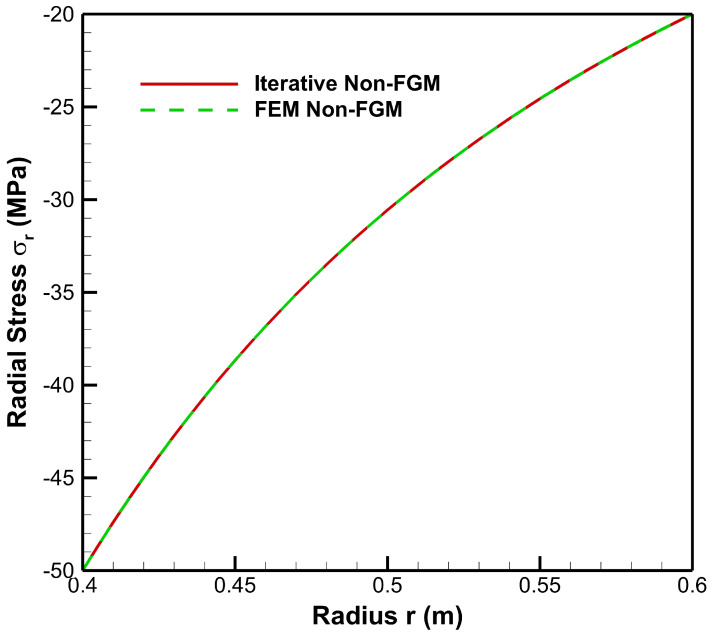
Comparison of radial stress along the radius of the cylinder under internal pressure, $P_{a}=50$ MPa and external pressure, $P_{b}=20$ MPa.

**Figure 17:**
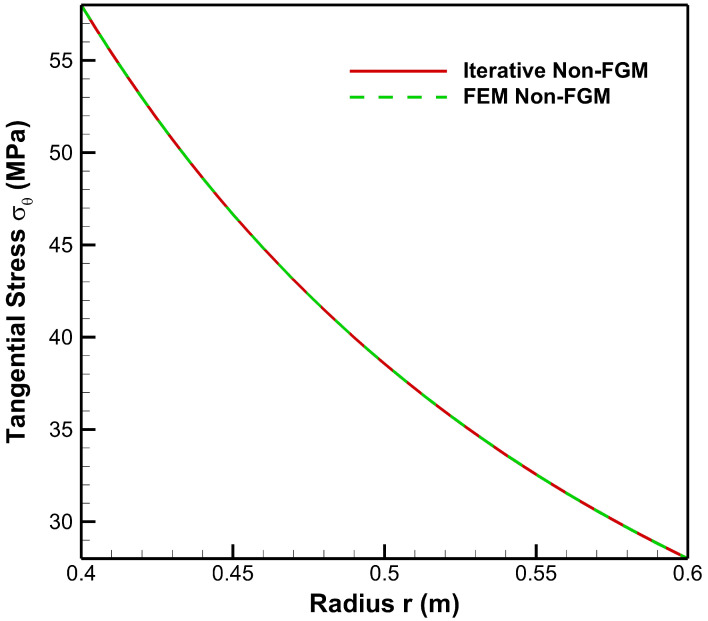
Comparison of tangential stress along the radius of the cylinder under internal pressure, $P_{a}=50$ MPa and external pressure, $P_{b}=20$ MPa.

Figure 16 and Figure 17 show a good agreement between iterative and finite element methods for the results of radial stress and tangential stress, respectively, in the case of the non-FGM cylinder.

**Figure 18:**
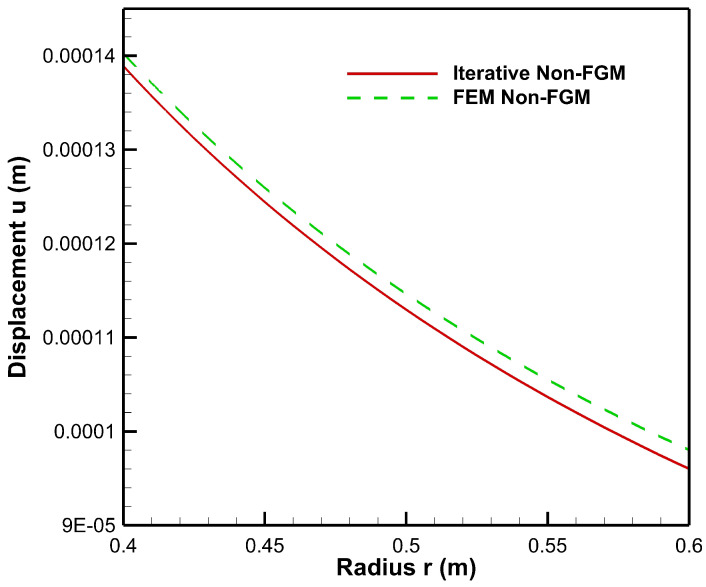
Comparison of displacement along the radius of the cylinder under internal pressure, $P_{a}=50$ MPa and external pressure, $P_{b}=20$ MPa.

From Figure 18, it is observed that finite element method solution for radial displacement in non-FGM cylinder varies with iterative solution only at fifth position after decimal.

Table 4 represents radial displacement in tabular form for different iteration stages a n=0, n=1 and n=2. In this table, $u_{0}$, $u_{1}$, and $u_{2}$ show initial, first and second iterations for radial displacement, respectively. From this table, it can be observed that relative error (%) is approximately less than and equal to 1% as we iterate from $u_{1}$ to $u_{2}$. The minimum relative error of 0.78% occurs at internal surface (HCS) of the cylinder and the maximum relative error of 1% occurs at external surface (MgO) of the cylinder.

Table 5 shows radial stress values in tabular form for the considered number of iterations in the study. It can be noted that relative error from the first iteration to the second iteration is much less (<0.5 %). It can also be seen that radial stress has zero error at the inner and outer radii of the cylinder. Tabular values of tangential stress for different iterations are presented in Table 6. From the table, it is found that relative error (%) from first iteration to second iteration is 1.5%We can see from Table 4, Table 5 and Table 6 that we have an overall relative error of less than 1.5% including error for radial displacement, radial stress and tangential stress, thus the obtained results of the iterative method are acceptable and are in good agreement with the solution obtained using the element method.

**Figure 19:**
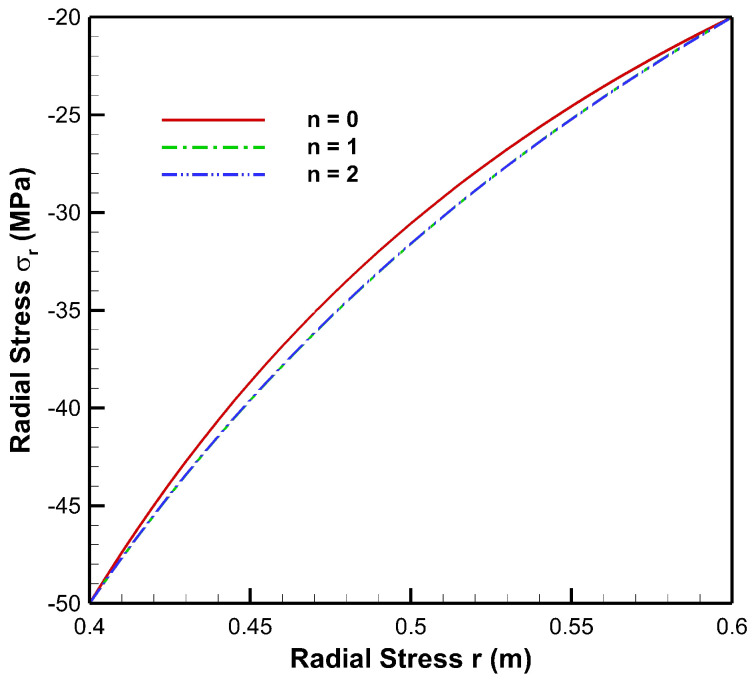
Iterative radial stress $\sigma_{\theta }$ in FGM cylinder at n=0, n=1 and n=2 iterations under internal pressure, $P_{a}=50$ MPa and external pressure, $P_{b}=20$ MPa.

**Figure 20:**
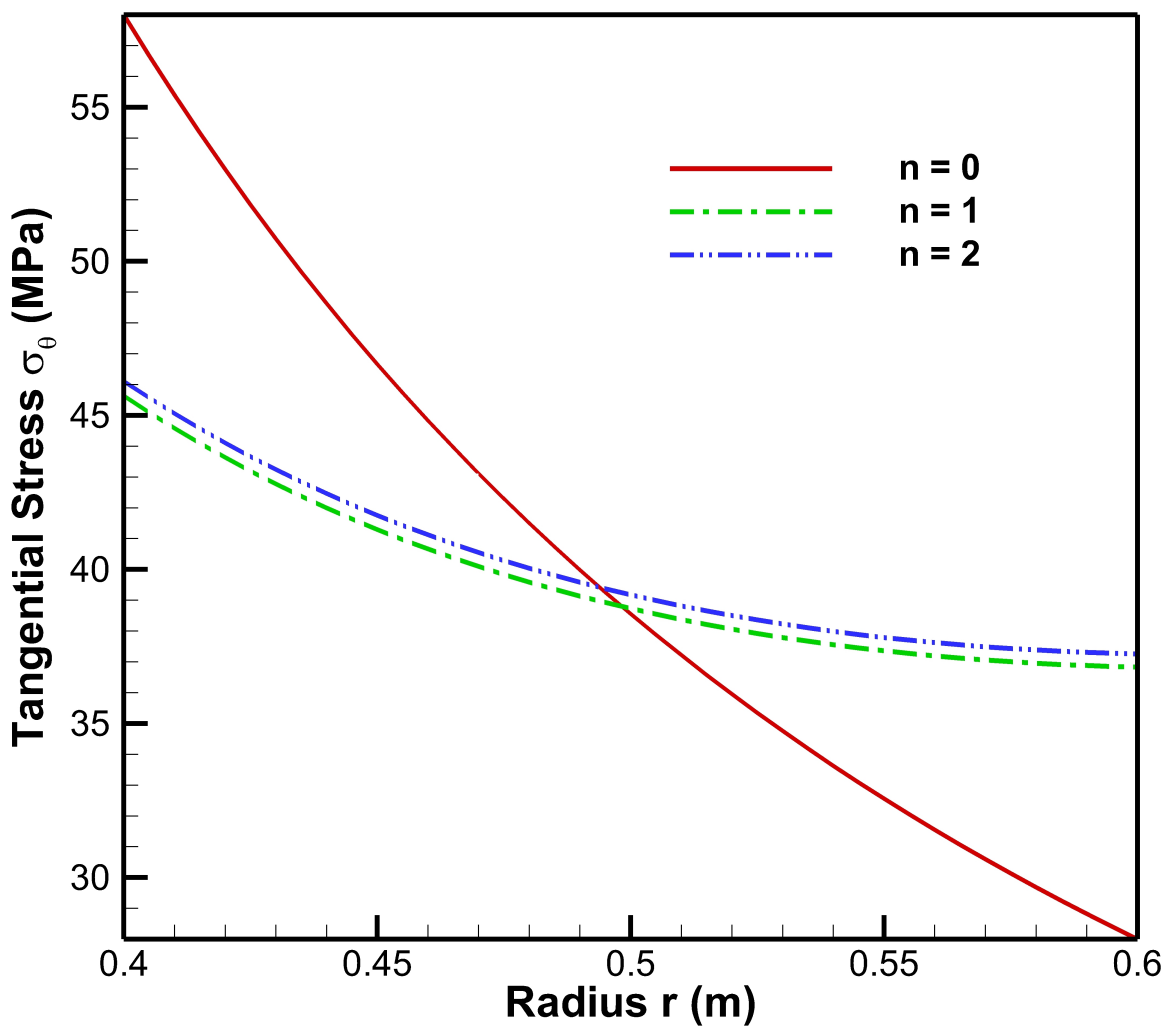
Iterative tangential stress $\sigma_{\theta }$ in FGM cylinder at n=0, n=1 and n=2 iterations under internal pressure, $P_{a}=50$ MPa and external pressure, $P_{b}=20$ MPa.

**Figure 21:**
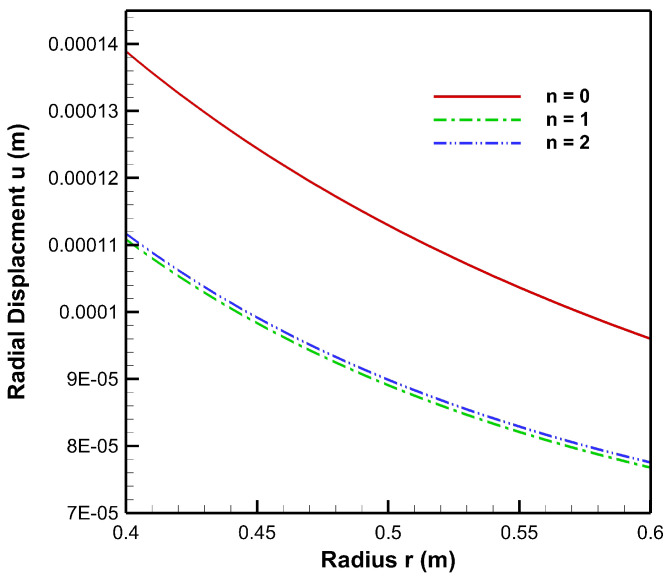
Iterative displacement $\sigma_{\theta }$ in FGM cylinder at n=0, n=1 and n=2 iterations under internal pressure, $P_{a}=50$ MPa and external pressure, $P_{b}=20$ MPa.

Figure 19, Figure 20 and Figure 21 present the behaviour of stresses and the displacement in the FGM cylinder under internal pressure ($P_{a}=50$ MPa)–external pressure ($P_{b}=20$) MPa for n=0, n=1 and n=2 iteration steps. Examining Figure 19 for the radial stresses obtained at different iterations, it can be noted that solution values of radial stress are refined at the second iteration, n=2. Moreover, in the case of tangential stress, Figure 20 can be observed to understand the efficacy of the iterative technique as tangential stress values obtained at first and second iterations have an average relative error of 1.13%. A similar outcome of the solution refinement can also be observed for displacement solution values from Figure 4, in which first and second iterations generate displacement values with 0.91% average relative error between them.

## 4. Conclusions

This study presents an implementation of a novel semi-analytical iterative technique to obtain displacement and stresses for an axisymmetric exponentially graded cylinder under internal and external pressure conditions. The novelty or originality of our work is demonstrated by applying an iterative technique to Navier’s equation under plain strain conditions. Further, results obtained are compared with the finite element method. For cylindrical pressure vessels, this iterative technique has not been implemented previously for steady state elastic stress analysis under exponential gradation. Moreover, the mechanical response of the FG cylinder made up of a High Carbon Steel (HCS) metal matrix and magnesium oxide (MgO) ceramic has not been observed in any such previous studies. The results show a good agreement between the iterative method and FEM, with an acceptable error which can be further improved by considering a greater number of iterations. Moreover, results for the HCS material have been validated using both methods. The analysis shows that the FGM cylinder made up of HCS (metal)–MgO (ceramic) with an exponential gradation is more capable of reducing stresses than the non-FGM (HCS) cylinder. The significant effect of the assumed in-homogeneity parameter can be seen in the mechanical responses of the cylinder.

## Figures and Tables

**Table 1:** Comparison of radial displacement in FGM cylinder using iterative technique and FEM under internal pressure, $P_{a}=50$ MPa and external pressure, $P_{b}=20$ MPa.

Radial Points	Iterative Radial Displacement	FEM Radial Displacement	Relative Error%
0.4	0.000111702	0.0001148556	2.82
0.425	0.000104959	0.0001080531	2.95
0.45	0.0000991793	0.0001022370	3.08
0.475	0.0000942017	0.0000972353	3.22
0.5	0.0000898958	0.0000929170	3.36
0.525	0.0000861567	0.0000891788	3.51
0.55	0.0000828990	0.0000859378	3.67
0.575	0.0000800525	0.0000831264	3.84
0.6	0.0000775589	0.0000806862	4.03

**Table 2:** Comparison of radial stress in FGM cylinder using iterative technique and FEM under internal pressure, $P_{a}=50$ MPa and external pressure, $P_{b}=20$ MPa.

Radial Points	Iterative Radial Stress	FEM Radial Stress	Relative Error%
0.4	−50	−49.98033938	0.04
0.425	−44.4208	−44.39839306	0.05
0.45	−39.5811	−39.63147769	0.13
0.475	−35.3391	−35.49674154	0.45
0.5	−31.5859	−31.80130361	0.68
0.525	−28.2363	−28.47351941	0.84
0.55	−25.2232	−25.4098732	0.74
0.575	−22.4924	−22.58186799	0.40
0.6	−20	−19.99327262	0.03

**Table 3:** Comparison of tangential stress in FGM cylinder using iterative technique and FEM under internal pressure, $P_{a}=50$ MPa and external pressure, $P_{b}=20$ MPa.

Radial Points	Iterative Tangential Stress	FEM Tangential Stress	Relative Error%
0.4	46.1103	47.9471693	3.98
0.425	43.6634	45.30832273	3.77
0.45	41.7588	43.18937037	3.43
0.475	40.2903	41.54299728	3.11
0.5	39.1781	40.26203588	2.77
0.525	38.361	39.31751127	2.49
0.55	37.792	38.64074181	2.25
0.575	37.4346	38.21511989	2.09
0.6	37.2599	37.97738751	1.93

**Table 4:** Comparison of radial displacement $u_{n}$ in FGM cylinder at n=0, n=1 and n=2 iterations under internal pressure, $P_{a}=50$ MPa and external pressure, $P_{b}=20$ MPa.

Radial Points	Iterative Radial Displacement *u* at n=0	Iterative Radial Displacement *u* at n=1	Iterative Radial Displacement *u* at n=2	Relative Error%
0.4	0.000138899	0.000110838	0.000111702	0.78
0.425	0.000131225	0.000104109	0.000104959	0.82
0.45	0.000124432	0.000098342	0.000099179	0.85
0.475	0.000118382	0.000093376	0.000094202	0.88
0.5	0.000112961	0.000089081	0.000089896	0.91
0.525	0.000108082	0.000085354	0.000086157	0.94
0.55	0.000103669	0.000082108	0.000082899	0.96
0.575	0.000099663	0.000079272	0.000080053	0.98
0.6	0.000096011	0.000076789	0.000077559	1.00

**Table 5:** Comparison of radial stress $\sigma_{r}$ in FGM cylinder at n=0, n=1 and n=2 iterations under internal pressure, $P_{a}=50$ MPa and external pressure, $P_{b}=20$ MPa.

Radial Points *r*	Iterative Radial Stress $\sigma_{r}$ at n=0	Iterative Radial Stress $\sigma_{r}$ at n=1	Iterative Radial Stress $\sigma_{r}$ at n=2	Relative Error%
0.4	−50	−50	−50	0.00
0.425	−43.8339	−44.4415	−44.4208	0.05
0.45	−38.6667	−39.6078	−39.5811	0.07
0.475	−34.2936	−35.3617	−35.3391	0.06
0.5	−30.56	−31.5984	−31.5859	0.04
0.525	−27.3469	−28.2371	−28.2363	0.00
0.55	−24.562	−25.2149	−25.2232	0.03
0.575	−22.1323	−22.4822	−22.4924	0.05
0.6	−20	−20	−20	0.00

**Table 6:** Comparison of tangential stress $\sigma_{\theta }$ in FGM cylinder at n=0, n=1 and n=2 iterations under internal pressure, $P_{a}=50$ MPa and external pressure, $P_{b}=20$ MPa.

Radial Points *r*	Iterative Tangential Stress $\sigma_{\theta }$ at n=0	Iterative Tangential Stress $\sigma_{\theta }$ at n=1	Iterative Tangential Stress $\sigma_{\theta }$ at n=2	Relative Error%
0.4	58	45.6366	46.1103	1.04
0.425	51.8339	43.195	43.6634	1.08
0.45	46.6667	41.2971	41.7588	1.12
0.475	42.2936	39.8365	40.2903	1.14
0.5	38.56	38.7326	39.1781	1.15
0.525	35.3469	37.9234	38.361	1.15
0.55	32.562	37.3607	37.792	1.15
0.575	30.1323	37.0064	37.4346	1.16
0.6	28	36.83	37.2599	1.17

## Data Availability

Not applicable.

## Abbreviations

The following abbreviations are used in this manuscript:

FGM	Functionally Graded Materials
FEM	Finite Element Method
